# Relationship between the oral cavity and cardiovascular diseases and metabolic syndrome

**DOI:** 10.4317/medoral.19563

**Published:** 2013-10-13

**Authors:** Esther Carramolino-Cuéllar, Inmaculada Tomás, Yolanda Jiménez-Soriano

**Affiliations:** 1 Degree in Dental Surgery. Grant fellow (FPU) of the Spanish Ministry of Education. Faculty of Medicine and Odontology. University of Valencia; 2 Assistant Professor of Stomatology. Faculty of Medicine and Odontology. University of Santiago de Compostela, Spain; 3 Assistant Professor of Stomatology. Faculty of Medicine and Odontology. University of Valencia, Spain

## Abstract

The components of the human body are closely interdependent; as a result, disease conditions in some organs or components can influence the development of disease in other body locations. The effect of oral health upon health in general has been investigated for decades by many epidemiological studies. In this context, there appears to be a clear relationship between deficient oral hygiene and different systemic disorders such as cardiovascular disease and metabolic syndrome. The precise relationship between them is the subject of ongoing research, and a variety of theories have been proposed, though most of them postulate the mediation of an inflammatory response. This association between the oral cavity and disease in general requires further study, and health professionals should be made aware of the importance of adopting measures destined to promote correct oral health. The present study conducts a Medline search with the purpose of offering an update on the relationship between oral diseases and cardiovascular diseases, together with an evaluation of the bidirectional relationship between metabolic syndrome and periodontal disease. Most authors effectively describe a moderate association between the oral cavity and cardiovascular diseases, though they also report a lack of scientific evidence that oral alterations constitute an independent cause of cardiovascular diseases, or that their adequate treatment can contribute to prevent such diseases. In the case of metabolic syndrome, obesity and particularly diabetes mellitus may be associated to an increased susceptibility to periodontitis. However, it is not clear whether periodontal treatment is able to improve the systemic conditions of these patients.

** Key words:**Cardiovascular diseases, periodontitis, metabolic syndrome, obesity, diabetes mellitus.

## Relationship between the oral cavity and cardiovascular diseases and metabolic syndrome

The human body is a system of components characterized by an intense interrelation of biological processes. Alterations of any of its parts have an impact upon other areas. As an example, inflammation may link oral diseases such as periodontitis to systemic disorders such as arteriosclerotic cardiovascular disease or metabolic syndrome (1).

In the last few decades these associations have been established on the basis of scientific evidence gained from epidemiological studies. From the first descriptions, the publications found in the literature have gradually increased in number, in an attempt to clarify the possible correlations, particularly in view of the high incidence and importance of the disease conditions involved. Nevertheless, there is still considerable uncertainty regarding the existing associations, the underlying etiopathogenic mechanisms, and the results obtained following the treatment of one condition or other (2).

The present review offers an update on the relationship between oral diseases and cardiovascular diseases, together with an evaluation of the bidirectional relationship between metabolic syndrome and periodontal disease.

## Material and Methods

A Medline-PubMed literature search was made, using the following descriptors or key words: “cardiovascular diseases”, “periodontitis”, “periodontal diseases”, “oral health”, “metabolic syndrome”. Under cardiovascular diseases, many articles include the terms “coronary heart disease”, “stroke”, “peripheral vascular disease”. Metabolic syndrome in turn comprises “obesity”, “diabetes mellitus”, “hyperglycemia”.

The search limits were publications in the last 10 years, in Spanish and English.

## Relationship between the oral cavity and cardiovascular diseases

Arteriosclerotic vascular disease or cardiovascular disease (CVD) affects the heart and blood vessels, and fundamentally comprises ischemic heart disease, cerebrovascular disease and peripheral vascular disease (International Classification of Diseases 9th Revision [ICD-9]. It is a progressive, chronic condition that can give rise to acute coronary ischemia (chest pain or angina), myocardial infarction or stroke (2,3).

The World Health Organization (WHO) cites ischemic heart disease and stroke as the leading causes of death in the industrialized world (12.2% and 9.7%, respectively). Aging of the population and the increase in risk factors likewise cause an increase in the prevalence of both diseases. Periodontal disease is also very common, with a prevalence that depends upon the age interval considered, ranging from 1% in patients between 20-29 years of age to 39% among those over 65 years of age.

In 1980, Simonka *et al.* related CVD to periodontitis, noting that patients with myocardial infarction had a higher prevalence of periodontal disease. Since then, many studies have examined this association (3).

Both periodontitis and myocardial infarction are of multifactorial origin, and involve a series of risk factors – some of which are common to both diseases. Cardiovascular disease has well defined risk factors (known as classical risk factors), such as dyslipidemia, hypertension, smoking, excess body weight, a sedentary lifestyle and diabetes. In general, the terms coronary risk and cardiovascular risk are used indistinctly. The term cardiovascular risk factors was introduced from follow-up observations of the Framingham cohort, and mathematical formulas have been developed to calculate the global risk. These classical risk factors are not always seen to be more prevalent in patients with cardiovascular diseases; as a result, new risk factors have been identified, based on etiopathogenic studies and supported by clinical, epidemiological and laboratory studies. Recently, chronic inflammation has been implicated in the etiology of cardiovascular disease, and epidemiological studies have shown inflammatory markers to be related to endothelial dysfunction, which is observed in the early stages of the atherogenic process. As a result, attempts have been made to determine whether periodontal disease, as a chronic inflammatory disorder, constitutes an independent risk factor for cardiovascular disease (4). The importance of such risk factors is explained by the fact that they can help to predict the probability of cardiovascular and/or cerebrovascular disease, and may contribute to the development of appropriate preventive and interventional strategies.

The pathogenic mechanism relating the two disorders has not been clearly established. Some authors point to the increase in inflammatory activity in periodontitis as the key mediating element, contributing to development of the atheroma plaque that causes the cardiovascular disease (5-7). Another linking mechanism could be cross-reactivity between antibodies against periodontal microorganisms and heat shock proteins such as HSP60 in the endothelial cells. These proteins are related to atheroma plaque formation (3). On the other hand, it has been postulated that bacteremia of periodontal origin may have a direct effect, since the lipopolysaccharides of these microorganisms cause macrophage stimulation and the release of inflammatory mediators (IL-1α and -1ß and TNF) that play an important role in atherogenesis. This periodontal flora may also induce platelet aggregation, thereby increasing the risk of thromboembolic phenomena (3).

The different studies relating the two diseases have been very diverse and have made use of different methodologies; as a result, comparisons are sometimes difficult to establish.

The prevalence of periodontitis in epidemiological studies is determined using clinical indexes such as the plaque index, gingival bleeding, pocket depth and attachment loss, or using oral health questionnaires. Direct microbiological markers have also been studied, such as the detection of periodontal microorganisms, as well as indirect markers such as the serum concentration of IgG or IgA antibodies targeted to such organisms. In turn, cohort studies have estimated the prevalence of periodontitis based on tooth loss – though this variable has an important limitation, since tooth loss can be caused by other oral diseases apart from periodontitis.

Cardiovascular diseases have been investigated in the context of observational clinical studies of patients with coronary disease and arteriosclerosis, with coronary ischemia or acute infarction, and individuals with cerebrovascular stroke. Complementary techniques have also been used to detect patients at risk, such as computed tomographic imaging of the coronary arteries, mag-netic resonance imaging, echodoppler ultrasound evaluation of the supraaortic trunks, or measurement of the ankle-brachial index. Noninvasive studies of the vascular endothelium are presently used to identify patients at risk or with subclinical atherosclerotic disease, based on the measurement of carotid media-intima thickness (IMT), the study of flow-mediated brachial artery dilatation to assess endothelial function, and the analysis of pulse wave velocity (PWV) to explore the elastic properties of the arteries. The different inclusion criteria used in application to patients with cardiovascular disease have also contributed to the variability of the results obtained (3,8).

As can be seen, the existing publications are broad and relate periodontal and cardiovascular disease using different study methods and population groups. Those studies focusing on populations at risk have found increased carotid media-intima thickness as a marker of atherosclerosis to be related to periodontal disease, particularly in patients with advanced periodontal disease (3,9,10). Polymerase chain reaction (PCR)-based studies of the periodontal microorganisms in subgingival plaque have related the presence of *Prevotella gingivalis* and *Prevotella nigrescens* to an increased carotid media-intima thickness as calculated by carotid duplex ultrasound. Other studies have reported a significant direct relationship between IgG antibodies against periodontal microorganisms and carotid media-intima thickness increments of ≥ 1 mm (3,11).

A relationship has also been established between inflammation and endothelial dysfunction, with alterations in vasodilatation. The association between periodontitis and endothelial dysfunction has been observed in different studies evaluating endothelial response in patients without known cardiovascular disease and with or without periodontitis - a significant decrease in endothelial response being recorded in the patients with severe periodontitis. Prospective follow-up studies in turn have described an association between periodontal treatment and improvement in vascular endothelial response (12).

The presence of a chronic systemic inflammatory condition has also been explored in patients with periodontitis. These individuals have been seen to have elevated blood inflammatory mediators related to cardiovascular disease, such as high sensitivity C-reactive protein (hsCRP), fibrinogen and IL-6 (9,13). The metaanalysis published by Paraskevas et al. concluded that there is strong evidence from cross-sectional studies that plasma C-reactive protein is increased in patients with periodontitis (> 2.1 mg/l), and these levels moreover decrease with periodontal treatment (14). Other inflammatory markers have also been found to be elevated in patients of this kind, though to a lesser degree. Increased levels of low-density lipoprotein cholesterol and its oxidation products (OxLDL) are regarded as risk markers of cardiovascular disease, and both have been shown to be increased in the crevi-cular fluid of patients with periodontitis (15).

Other studies relating the two disease conditions have evaluated the participation of periodontal microbiological parameters in arteriosclerosis, based on the isolation and identification of oral pathogens (*Actinobacillus actinomycetemcomitans [Aggregatibacter], Porphyromona gingivalis, Streptococcus mutans*) in atheroma plaques (3,16). Not all the studies have been able to isolate these pathogens in atheroma plaques; nevertheless, PCR techniques have detected the presence of bacterial DNA (3), though in some cases it has not been possible to confirm that this DNA is specific of periodontal bacteria.

Experimental studies have been made in animals and cell cultures with the purpose of evaluating how vascular invasion by and exposure to periodontal pathogens such as *Porphyromonas gingivalis* contribute to atheroma plaque formation. In cell cultures, such exposure has been related to an increase in cytokine production, increased monocyte recruitment and activation, and the formation of foam cells. All these phenomena contribute to atheroma plaque formation. In experimental studies in animals, vascular invasion by *Porphyromonas gingivalis* has been related to an increase in smooth muscle proliferation markers (S100A9, SMemb), and to the induction of hyperplasia of the tunica intima of the aorta (3).

In further support of the relationship between periodontal disease and cardiovascular disease, Machuca *et al.* conducted a 10-year longitudinal prospective study in which patients with coronary disease were found to have a poorer periodontal condition than patients without coronary disease, and moreover responded less favorably to periodontal treatment (17). One of the most controversial issues is whether periodontal treatment modifies the risk of developing arteriosclerotic vascular disease or its complications. The data found in the literature are not all homogeneous or concordant in this respect. Some studies have observed improvements in subclinical arteriosclerosis markers such as endothelial function after periodontal treatment; however, the results are highly variable when systemic inflammatory markers are considered. Kamil *et al.* (18) found that the nonsurgical treatment of periodontal disease in patients with coronary disease or cardiovascular risk factors significantly reduces the serum concentration of C-reactive protein (18) and the concentration of atheromatous lipids (LP-pu, LA2, Li, oxidized-DL) in patients with dyslipidemia. These improvements vary over time, and are generally observed after 6 months of treatment – though a return to baseline levels is observed after one year (3). Paraskevas *et al.* (14) conducted a systematic review and metaanalysis of the changes in serum C-reactive protein concentration following periodontal treatment, and concluded that the levels of this inflammatory marker decrease slightly after treatment, with a pondered average reduction of 0.50 mg/l; (95%CI: 0.08-0.9)(*p*=0.02) (14).

Due to the large number of published studies with often contradictory results, metaanalyses have been carried out in an attempt to establish a degree of scientific evidence of the association between the two diseases. In this context, the metaanalysis published by Bahekar *et al.* in 2007 identified a statistically significant relationship between periodontal disease and coronary disease, in terms of both incidence and prevalence. However, the authors underscored that the association between the two conditions was moderate (with an odds ratio [OR] of < 2). Specifically, the risk of suffering coronary disease was found to be 1.24-fold greater in edentulous individuals (with < 10 teeth), and an inverse correlation was observed between the number of teeth and the risk of coronary disease. In any case, the authors also pointed out that the partially edentulous population has poorer oral hygiene, a high carbohydrate intake in the diet, and an important number of smokers (5). In 2008, Humphrey *et al.* (19) carried out a metaanalysis of the periodontal condition (including gingivitis, bone loss and tooth loss) in the general population (i.e., not limited to patients with cardiovascular disease), and concluded that periodontal disease appears to be an independent but weak risk factor for coronary disease.

In sum, there is great variability among the studies that have explored the association between oral alterations and cardiovascular disease – most authors reporting a moderate association, without the firm scientific evidence needed to confirm a true causal relationship (8).

The confirmation of periodontal disease as an independent risk factor would result in more aggressive treatment of patients at a high risk of developing cardiovascular disease. More solid scientific evidence is required in this respect, based on longitudinal studies with standardized measurements and prolonged follow-up, controlling for other risk factors, and randomized studies of periodontal treatment.

## Relationship between metabolic syndrome and periodontitis: Diabetes mellitus and obesity

Metabolic syndrome (MetS), according to the International Diabetes Federation (IDF), is a complex series of symptoms defined by the presence of three or more of the five characteristic MetS components: hypertension, hypertriglyceridemia, low HDL-cholesterol, obesity and elevated blood glucose (or type 2 diabetes mellitus) (20).

Many epidemiological studies have examined the relationship between oral health and different systemic conditions such as metabolic syndrome (21-23). At present, research focuses on the identification of possible mechanisms underlying this association, and on establishing whether the treatment of oral disease leads to improvement in the systemic disease markers (24,25). Although there is much controversy in this field, most authors agree that both disease conditions share a common physiopathological mechanism, associated with the existence of systemic inflammation and insulin resistance (26).

D’Aiuto *et al.* (22), in a study of 13,170 individuals in the Third National Health and Nutrition Examination Survey (NHANES III), reported a direct relationship between periodontitis and metabolic syndrome in people over 45 years of age. Likewise, Morita *et al.* (23), in 1023 patients presenting all the metabolic syndrome components within normal limits at the start of the study, found the presence of periodontal pockets after four years of follow-up to be associated with positive conversion of one or more of the metabolic syndrome components. This correlation in turn was seen to be greater among elderly individuals, males and smokers, thus suggesting that the prevention and treatment of periodontitis could lessen the risk of developing metabolic syndrome. A recent metaanalysis published by Nibali *et al.* (21) has revealed a clear association between metabolic syndrome and periodontitis – the subjects with metabolic syndrome being almost twice as likely to develop periodontitis than the rest of the population.

Diabetes

The relationship between diabetes and periodontal disease has been widely examined in the literature, and is taken as an example of how a systemic disorder can predispose to oral infections, and of how the latter in turn can exacerbate systemic disease (24,27-29). Specifically, a number of studies have shown diabetes mellitus to be a risk factor for periodontitis (29,30).

Hyperglycemia may be the main cause of diabetic complications by inducing an increase in the production of advanced glycosylation end-products (AGEs) secondary to the non-enzymatic glycosylation of proteins and lipids. The physiopathological relationship between diabetes and periodontal disease is explained by the capacity of both disorders to induce an inflammatory response through AGEs or bacterial accumulation, respectively – thereby giving rise to the production of inflammatory mediators (31).

Different cells, such as endothelial cells or phagocytes, present AGE receptors (RAGE) at surface level. In this context, AGE/RAGE binding upon mononuclear or polymorphonuclear cells (PMNs) inhibits chemotaxis and the phagocytic capacity of these cells, allowing the proliferation of gramnegative bacteria. As a result of this mechanism, macrophages and PMNs show hyperresponsiveness to bacterial antigens, with an increased release of cytokines such as IL-1β and TNF-α, and of inflammatory mediators – this giving rise to increased periodontal destruction. Furthermore, alteration of the fibroblast population would induce changes in collagen production, giving rise to wound healing problems and contributing to the development of periodontal disease. This in turn would be aggravated by an increase in the activity of collagenase and of other enzymes at connective tissue level (29,32).

In the same way that diabetes can affect oral and periodontal health, periodontal inflammation may exert a negative effect upon the metabolic control of diabetes as a result of the release of inflammatory mediators such as TNF-α, which would act upon the cellular insulin receptors, thereby complicating insulin action. In this context, lipopolysaccharides can aggravate diabetes through an increased production of IL-1β, TNF-α and PGE2. In turn, bacterial infections can reduce glucose uptake on the part of the skeletal muscle, thereby also giving rise to insulin resistance (24). In fact, a study analyzing the relationship between the severity of periodontal disease and blood glucose control concluded that those patients with a lesser percentage of locations with bleeding upon probing and pockets measuring ≥ 5 mm in depth presented better blood glucose control than those patients with poorer periodontal conditions (33).

The effect which periodontal treatment may exert upon the metabolic control of diabetes is the subject of debate. Some authors such as Kiran et al. (34) have described a statistically significant decrease in the glycosylated hemoglobin (HbA1c) levels in diabetic patients after periodontal treatment. However, Janket *et al.* (25), in a metaanalysis of 456 patients with type 1 and type 2 diabetes mellitus reported a 0.38% decrease in HbA1c – the decrease being greater (0.71%) in those patients with type 2 diabetes receiving treatment combined with antibiotics. In neither case were the differences statistically significant. Studies involving a large sample size are needed in order to corroborate the benefits of periodontal treatment in the metabolic control of diabetes.

Obesity 

Recent studies have suggested that obesity could be associated to oral diseases, particularly periodontitis (35-37). The causal relationship between obesity and periodontitis, and the possible underlying biological mechanisms, have not been clearly established. However, adipose tissue is known to secrete a range of hormones and cytokines that contribute to systemic inflammation and insulin resistance – thus suggesting that there are physiopathological similarities between obesity and periodontitis, which in turn could lead to the development of type 2 diabetes and cardiovascular diseases (37,38). The most important adipocyte cytokines are leptin and adiponectin, though there are also other molecules such as resistin (37,39). Leptin exerts a protective effect against obesity and is related to bone metabolism. It has been suggested that elevated leptin levels can be found during infection and inflammation, though the role of this molecule has not been firmly established. Adiponectin in turn improves insulin sensitivity and is found to be decreased in obese people. It is thought to possibly act as an inflammatory mediator. Resistin in turn exhibits a potent proinflammatory effect (37).

However, many investigators point to TNF-α, which is abundantly expressed in the adipose tissue of obese individuals, as the most important mediator related to obesity and periodontitis. Increases in circulating TNF-α appear to exacerbate periodontal inflammation, and this may be one of the reasons why obese individuals are more susceptible to periodontal disease (40). Thus, an increase in TNF-α not only affects insulin sensitivity in obese subjects but also influences periodontal inflammation. Exacerbated periodontal inflammation in turn may further increase the concentration of TNF-α through the stimulation of monocytes, and may exert additive effects upon insulin resistance - directly influencing target organs such as the liver, muscles and adipocytes, or indirectly causing adipocyte lipolysis with the resulting release of free fatty acids, which also inhibit insulin action (39,40).

The existing scientific evidence suggests that obesity, and particularly diabetes mellitus, could be related to an increased suscepti-bility to periodontitis. However, it is not clear whether periodontal treatment could improve the systemic conditions of such patients.
